# The Surgical Congress: July 27-31

**Published:** 1914-05-23

**Authors:** 


### THE SURGICAL CONGRESS: JULY 27—31.

### Institutional Personalities.

The programme of addresses and discussions contains many noted and representative names. Sir Rickman Godlee, who opens with an address of welcome, is well known to the institutional world as surgeon, past or present, to University College Hospital, Charing Cross Hospital, the Brompton Consumption Hospital, and the Queen's Hospital for Children. He is, of course, also President of the Royal College of Surgeons, and nephew of Lord Lister. The new President, Dr. J. B. Murphy, of Chicago, who will then be inaugurated, is a surgeon of world-wide fame, not only as an original worker who has contributed much to the progress of surgery in the last twenty-five years, but also as one of the most celebrated surgical teachers of the day. By this double gift of originality and exposition Dr. Murphy has influenced the current surgical profession in America perhaps more than any single man has been able to do before. His presidential address will be upon bone transplantation; and on the same day Professor von Eiselsberg, of Vienna, will read a paper upon the still vexed question of the best method of operation for ulcer of the stomach. This paper will be discussed by Sir Watson Cheyne, who came to London from Edinburgh with Lister, and has been connected with the King's College Hospital staff ever since. On the second day Dr. E. W. Andrews, Chicago, will read a paper on "Tissue Inlaying" in the radical cure of hernia, a method which American surgeons have been using for some little time, but which is less familiar on this side of the Atlantic. Mr. McGavin, an old Guy's man, now on the staff of the *Dreadnought* Hospital, will discuss this paper: he is known as a strong advocate of filigree implantation, and is well qualified to carry on the debate initiated by Dr. Andrews. On the same day Mr. R. Jones, of Liverpool, will deal with an old topic in the treatment of certain derangements of the knee-joint. This celebrated orthopædist is one of the noted British leaders in his specialty, and his private workshops at Liverpool are one of the surgical sights which foreign visitors to England always insist on inspecting.

On the third day of the Congress (July 29) the new Vice-President, Dr. G. E. Armstrong, of Montreal, will read a paper on typhoid perforation, to be followed by remarks from Sir Anthony Bowlby, the well-known St. Bartholomew's surgeon. Professor Tuffier, of Paris, will next deal with transplantation of the ovaries; his most widely known achievement is his pioneer work on spinal anæsthesia some years ago. Then Dr. Charles Mayo, of Rochester, U.S.A., will give his views on the results of operations for exophthalmic goitre: he is the younger of the two celebrated brothers who manage the Mayo clinic, justly termed the Mecca of the surgical world, and has had an enormous experience of the topic he has selected. His paper will be discussed by Mr. James Berry, of the Royal Free Hospital, undoubtedly the best authority in Britain, and certain to maintain the debate on the high level to which Dr. Mayo is sure to lift it.

On July 30 Professor Krönig, of Freiburg, will discuss the principles of the non-operative treatment of carcinoma; to be followed by Dr. J. F. Percy, Illinois, on treatment of inoperable carcinoma of the uterus by the application of heat. Mr. Wilson will deal with the radical operation for cancer in the same organ; he is an old University College Hospital man, who has been for many years a specialist in gynæcology in Birmingham, and holds important hospital appointments there. In the discussion there will take part Dr. T. W. Eden, of Charing Cross, Chelsea, and Queen Charlotte's Hospitals; Mr. W. E. Miles, of the Cancer and the Gordon Hospitals; and Dr. J. C. Bloodgood, of Baltimore, whose contributions to transatlantic surgical literature have been abstracted more than once in The Hospital.

On the concluding day of the Congress Dr. Henry Jellett, the Dublin obstetrician, will deal with certain factors in the treatment of uterine prolapse; his paper will be discussed by Dr. Herbert Spencer, formerly professor at University College Hospital. Intestinal stasis will then be debated by Sir William Osler, Professor of Medicine at Oxford University; Sir W. Arbuthnot Lane, the famous Guy's surgeon; and Dr. J. C. Bloodgood, already mentioned.

The gynæcologists, it will be seen, are allotted part of the time which is at the disposal of the general surgeons. The laryngologists, otologists, and ophthalmologists, on the other hand, have separate sessions of their own. On Tuesday, July 28, Professor Schmiegelow, from Copenhagen, will read a paper on the operative treatment of cancer of the larynx; and Sir StClair Thomson will discuss it. The latter is Professor of Laryngology at King's College Hospital, and, curiously enough, possesses also the title of *physician* for diseases of the throat there, a relic doubtless of the days before surgery dared to invade this region. Further, Dr. J. M. West, of Berlin, and Dr. D. R. Paterson, of Cardiff, will discuss certain aspects of the surgery of the tear-glands.

On July 29 the otologists have their opportunity, and papers in this subject will be contributed by four well-known British specialists: Dr. Logan Turner, Edinburgh; Mr. Sidney Scott, St. Bartholomew's, London; Mr. H. E. Jones, Liverpool; and Mr. Hunter Tod, the London Hospital. Next day it is the turn of the ophthalmologists. Lieut.-Col. R. H. Elliott, I.M.S., will explain his own trephining operation for glaucoma. The other speakers announced for the day are Mr. Treacher Collins, of Charing Cross and the Royal London Ophthalmic Hospitals; Mr. F. Richardson Cross, of the Bristol Royal Infirmary; Mr. N. B. Harman, of the West London Hospital; Mr. J. B. Story, Professor of Ophthalmic Surgery in Dublin; and Mr. Holmes Spicer, of St. Bartholomew's Hospital.

